# Novel technique for cognitive magnetic resonance imaging‐transperineal ultrasound fusion transperineal prostate biopsy with injection of bubbled jelly into urethra after abdominoperineal excision

**DOI:** 10.1002/iju5.12624

**Published:** 2023-08-14

**Authors:** Keiichiro Miyajima, Fumihiko Urabe, Juria Nakano, Kosuke Iwatani, Kojiro Tashiro, Shunsuke Tsuzuki, Shun Sato, Hiroyuki Takahashi, Takahiro Kimura, Kenta Miki

**Affiliations:** ^1^ Department of Urology The Jikei University School of Medicine Minato‐ku Tokyo Japan; ^2^ Department of Pathology The Jikei University School of Medicine Minato‐ku Tokyo Japan

**Keywords:** cognitive MRI‐TPUS fusion biopsy, prostate biopsy, rectum resection

## Abstract

**Introduction:**

Prostate biopsy is commonly performed using a transrectal ultrasound probe through a transrectal or transperineal approach. However, this is not possible for patients without a rectum.

**Case presentation:**

A 75‐year‐old male was referred to our hospital because of an elevated prostate‐specific antigen and a suspicious prostate lesion (PIRADS 5) in the left peripheral zone. The patient had previously undergone abdominoperineal resection for rectal cancer, which excluded the use of transrectal ultrasound. We describe the use of the transperineal ultrasound‐guided biopsy with cognitive magnetic resonance imaging‐transperineal ultrasound fusion and the utility of the injection of bubbled lidocaine jelly into urethra to improve its visualization. The pathological findings revealed clinically significant cancer with a Gleason score of 5 + 4.

**Conclusion:**

Cognitive magnetic resonance imaging‐transperineal ultrasound fusion transperineal prostate biopsy with injection of bubbled jelly into urethra is a feasible and practical technique that does not require any specialized equipment.

**Abbreviations & Acronyms**
CT: computed tomography
DWI: diffusion‐weighted imaging
MRI: magnetic resonance imaging
PSA: prostate‐specific antigen
TPUS: transperineal ultrasound

**Keynote message**

Cognitive MRI‐TPUS fusion transperineal prostate biopsy with injection of bubbled jelly into urethra was performed in a patient who previously underwent abdominoperineal excision and lacked a rectum.

## Introduction

Prostate biopsy is typically performed using a transrectal ultrasound probe through a transrectal or transperineal approach. However, this method is not feasible for patients without a rectum. Herein, we report a technique for TPUS‐guided prostate biopsy with cognitive MRI fusion.

## Case presentation

**Fig. 1:**
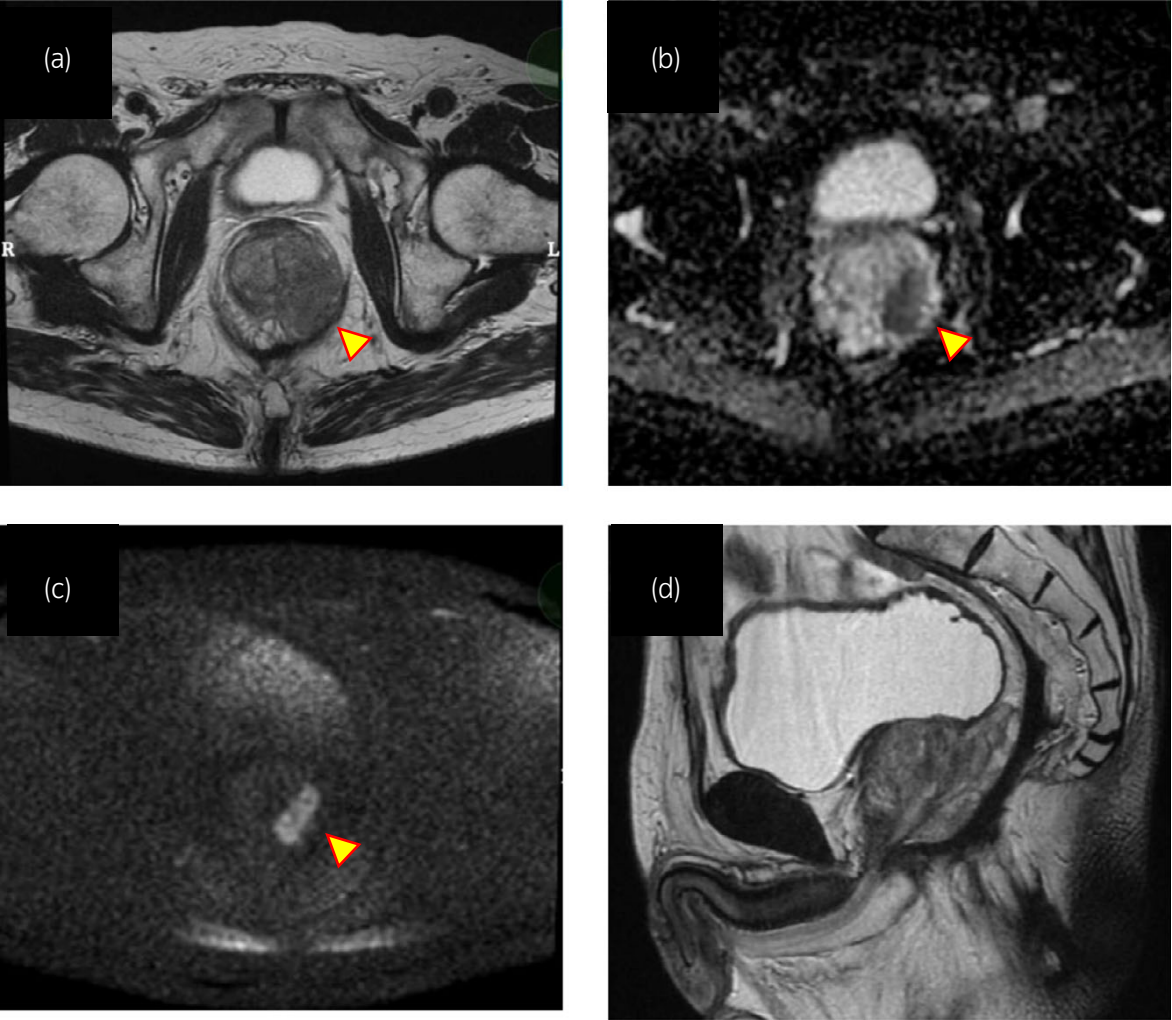
Imaging findings of the prostate. Multiparametric MRI demonstrated a well‐defined lesion in T2 (a), diffusion (b), and apparent diffusion coefficient (c) axial sections in the left peripheral zone, with overall features of a PIRADS 5 lesion. T2 sagittal section of prostate (d).

A 75‐year‐old male was referred to our hospital after routine health screening revealed an elevated serum level of PSA (5.85 ng/mL). Multi‐parametric MRI demonstrated a 25 cc gland with high signal intensity in T2‐weighted images, high signal intensity in DWI, and low signal intensity in apparent diffusion coefficient map images, indicating a PIRADS 5 lesion suggestive of prostate cancer (Fig. 1). The patient had a history of rectal cancer, treated with abdominoperineal excision >20 years prior. A transperineal prostate biopsy performed 3 months previously, with a PSA level of 4.5 ng/mL, did not show any malignancy. The patient was referred to our hospital for a re‐biopsy based on the elevated PSA level and the PIRADS 5 lesion.

**Fig. 2:**
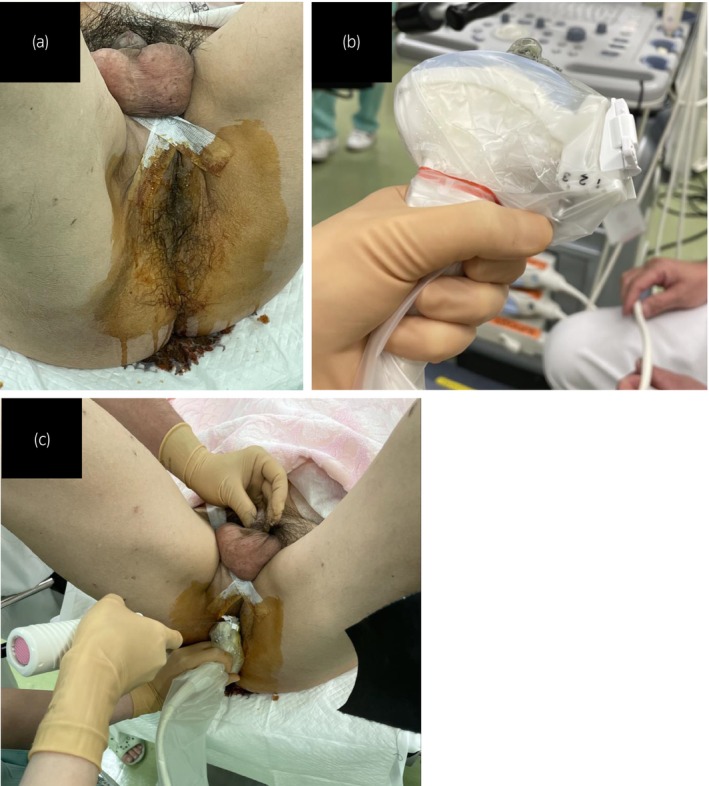
Transperineal approach for prostate biopsy. (a) The scrotum was retracted to expose the perineum. (b) TPUS scanning was performed using an abdominal ultrasound transducer with nephrostomy grid. (c) The biopsy gun was placed within the grid under ultrasound guidance, and prostate cores were obtained.

**Fig. 3:**
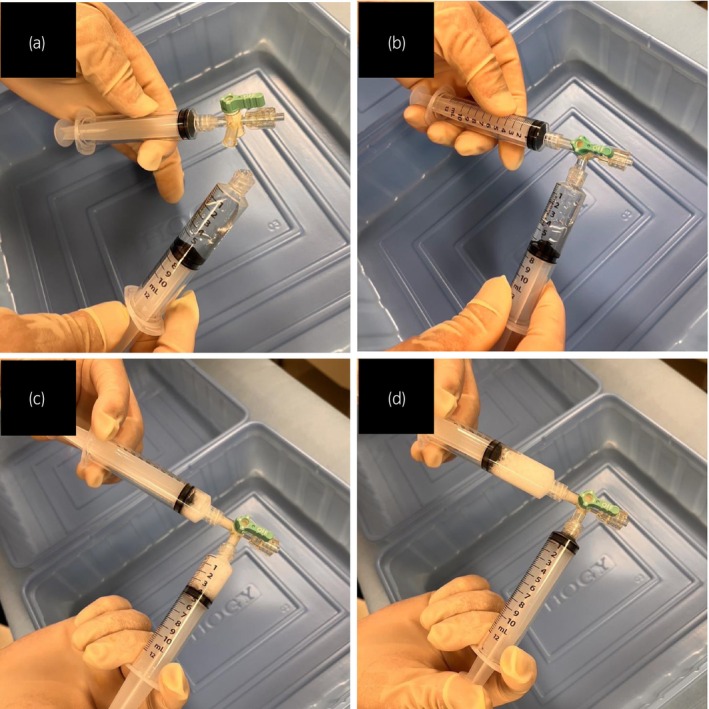
Bubbled jelly formation. (a) Combining 2 mL normal saline, 2 mL 2% lidocaine jelly, and 2 mL air in a syringe. (b) Connecting the two syringes with a three‐way stopcock. (c, d) Mixing the contents to make bubbled jelly.

**Fig. 4:**
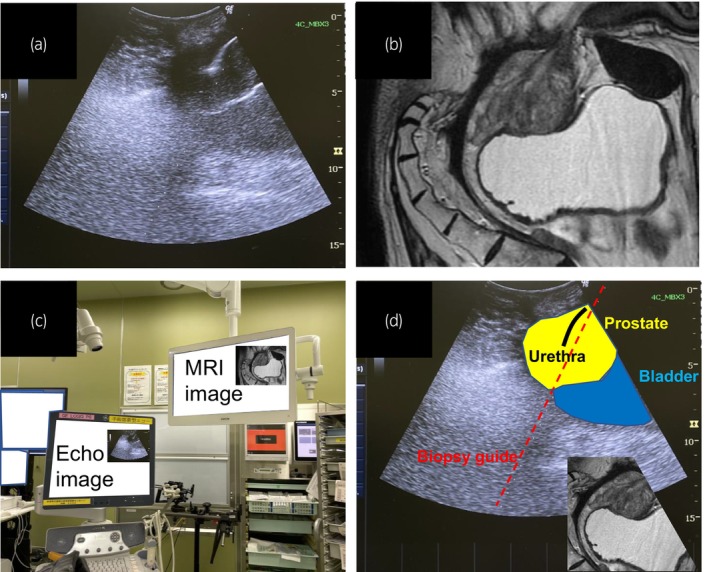
Cognitive MRI‐TPUS fusion biopsy. (a) TPUS scanning. The probe was placed vertically along the body to create a sagittal view. (b) Sagittal MRI of the prostate was flipped upside down. (c) The arrangement of devices to perform cognitive MRI‐TPUS fusion prostate biopsy. (d) Cognitive MRI‐TPUS fusion image.

Spinal anesthesia was administered and the patient was placed in the lithotomy position. The scrotum was retracted to expose the perineum (Fig. 2). TPUS was performed using an abdominal ultrasound transducer (4C‐RS probe, 21700BZY00451000; GE Healthcare, Chicago, IL, USA) with a nephrostomy grid (13B1X00150UP0037; GE healthcare). Biopsies were performed using an 18‐gauge biopsy needle. A novel strategy was used to facilitate the identification of biopsy needle insertion point, employing a 60° angle of approach in conjunction with the nephrostomy grid. We instilled 5 mL bubbled lidocaine jelly into the urethra to improve the visualization of urethra (Fig. 3). Although a urethral catheter is usually inserted, it could interfere with the visualization of the prostate in this case. Therefore, we opted for the lidocaine jelly. Indeed, we could not identify the tumor suspected area by TPUS, however, the ultrasound probe clearly detected the prostate, urethra, and bladder (Fig. 4). Then we inverted the MRI and placed the monitor next to the ultrasound machine. This allowed the operators to fuse the ultrasound image with the MRI image, and aided the identification and biopsy of suspected lesions. Finally, using the cognitive MRI‐TPUS fusion technique, multiple biopsies were obtained from the suspected lesion in the multiparametric MRI. Systemic prostate biopsies were also performed. The patient was discharged the day after the procedure, and experienced no postoperative complications.

Systemic prostate biopsies (six cores) and cognitive targeted MRI biopsies (two cores) for the left peripheral suspected lesion were performed. The biopsy specimens were analyzed by a genitourinary pathologist (SS). The prostatic biopsy revealed a Gleason score 9 (5 + 4) adenocarcinoma in two specimens (one systemic and one targeted biopsy).

## Discussion

The management of patients with elevated PSA who lack a rectum poses a challenge for urologists. The advent of community prostate cancer screening, recent advances in imaging, and improved survival of rectal cancer patients have increased the likelihood of encountering such patients. In this report, we detail a practical procedure for prostate biopsy in a patient without a rectum through cognitive MRI‐TPUS fusion. There are two important factors for performing an effective prostate biopsy in patients without rectal access. First, the insertion of bubbled lidocaine jelly into the urethra enables the ultrasonographic identification of the urethra and visualization of the prostate under the urethra. Second, the arrangement of the ultrasound machine and MRI enables the operator to obtain images in the same field of view.

In previous reports, prostate biopsies in patients without a rectum have been performed using various methods, including transabdominal ultrasound‐guided transperineal biopsy,^1^ transurethral ultrasound‐guided transperineal biopsy,^2^ and CT‐^3^ or MRI‐guided transgluteal biopsy.^4^ However, transabdominal ultrasound‐guided biopsies are associated with a risk of injury to the bowel and dorsal vein complex.^1^ Furthermore, transurethral ultrasound‐guided biopsies and CT‐ or MRI‐guided transgluteal biopsies may prove challenging in general hospitals. Recently, the efficacy of using an endocavity biplane transducer for transperineal prostate biopsy was reported in patients without a rectum.^5^ Nonetheless, its implementation in general hospitals would require a transperineal biopsy grid to ensure appropriate transducer positioning, which may pose a significant obstacle.

Previous reports have demonstrated the efficacy of novel procedures in improving the precision of prostate biopsies, albeit with potential challenges for implementation due to equipment limitations. However, an abdominal ultrasound transducer is a commonly available device in many hospitals. In the present case, it facilitated clear visualization of the prostate and safe completion of the biopsy procedure at a lower cost. On the other hand, in this case, we could not clearly identify the tumor suspected area by TPUS, which might be due to the use of the lower frequency (2–2.5 MHz) of the ultrasound transducer. The biplane transducer (5–10 MHz) would be better to identify the tumor suspected area in the prostate. This case highlights an interesting aspect of prostate cancer management in the MRI era for patients with a high pre‐test probability of cancer, in whom there are difficulties obtaining a tissue diagnosis. We hope that this approach may prove valuable to urologists in their daily clinical practice.

## Author contributions

Keiichiro Miyajima: Conceptualization; data curation; writing – original draft. Fumihiko Urabe: Conceptualization; data curation; resources; visualization; writing – original draft; writing – review and editing. Juria Nakano: Data curation; writing – review and editing. Kosuke Iwatani: Data curation; writing – review and editing. Kojiro Tashiro: Data curation; writing – review and editing. Shunsuke Tsuzuki: Data curation; supervision; visualization; writing – review and editing. Shun Sato: Data curation; supervision; visualization; writing – review and editing. Hiroyuki Takahashi: Conceptualization; supervision; writing – review and editing. Takahiro Kimura: Supervision; writing – review and editing. Kenta Miki: Conceptualization; data curation; supervision; writing – review and editing.

## Conflict of interest

The authors declare no conflict of interest.

## Approval of the research protocol by an Institutional Reviewer Board

Not applicable.

## Informed consent

Consent to participate and for publication were acquired from the patient.

## Registry and the Registration No. of the study/trial

Not applicable.
